# Pyrrhic victories: the need for social status drives costly competitive behavior

**DOI:** 10.3389/fnins.2013.00189

**Published:** 2013-10-23

**Authors:** Wouter van den Bos, Philipp J. M. Golka, David Effelsberg, Samuel M. McClure

**Affiliations:** ^1^Department of Psychology, Stanford UniversityStanford, CA, USA; ^2^Center for Adaptive Rationality (ARC), Max-Planck-Institute for Human DevelopmentBerlin, Germany; ^3^Department of Psychology, Heinrich-Heine-UniversityDüsseldorf, Germany; ^4^Department of Psychology, Ruhr-UniversityBochum, Germany

**Keywords:** competition, affect, social status, testosterone, cortisol, minimal groups

## Abstract

Competitive behavior is commonly defined as the decision to maximize one's payoffs relative to others. We argue instead that competitive drive derives from a desire for social status. We make use of a multi-player auction task in which subjects knowingly incur financial losses for the sake of winning auctions. First, we show that overbidding is increased when the task includes members of a rival out-group, suggesting that social identity is an important mediator of competitiveness. In addition, we show that the extent that individuals are willing to incur losses is related to affective responses to social comparisons but not to monetary outcomes. Second, we show that basal levels of testosterone predict overbidding, and that this effect of testosterone is mediated by affective responses to social comparisons. Based on these findings, we argue that competitive behavior should be conceptualized in terms of social motivations as opposed to just relative monetary payoffs.

## Pyrrhic victories: testosterone mediates costly competitive behavior

Two conflicting conceptualizations of competitive drive exist in the social science literature. First, in economics, competition is commonly defined as the desire to maximize one's payoffs relative to others (Messick and McClintock, 1968). This formulation underlies the social value orientation (SVO) measure of competitive drive that is broadly used to assay competitiveness (Murphy et al., 2011). However, this definition of competitive drive does not account for the fact that competition often leads to outcomes with *negative* absolute and relative payoffs. For example, when competing for items in auctions, people often bid far more than their estimated utility of the good (Ku and Malhotra, 2005). Consequently, winning the competition incurs net monetary losses while opponents' revenue remain unchanged.

The second conceptualization of competition considers it to be the dominant means for determining status within a hierarchy for both humans and animals (Sapolsky, 2004). Although social status is clearly associated with the ability to obtain power and resources (Lin, 1999), several studies have also suggested that individuals often consider status an end in itself (Barkow, 1989; Frank, 1993; Huberman et al., 2004). This is in line with classic research in economics linking the drive for status with the costly consumption of positional goods (Frank, 1993; Veblen, 2000). Evidence such as this leads to a view of competitive drive as motivation to obtain social outcomes independent of other considerations. Thus, behavior in competitive environments may not only be based on expected monetary outcomes but also on the utility ascribed to being the winner or loser.

The underlying hypothesis of this paper is that an intrinsic need for social status is an important driver of competitive behavior in economic decision-making, and, as a result, monetary losses can occur as long as there are offsetting social gains. To test this, we assess competitive drive using a common value auction paradigm in which the motivation to win (and avoid losing) can be measured on a continuous monetary scale. Specifically, the optimal bidding strategy in this paradigm is well-known (Kagel and Levin, 2009) and can be easily instructed to auction participants (van den Bos et al., 2008). One of the main advantages of the auction task is therefore that the degree to which (equilibrium) bids exceed the optimum serves as a direct quantitative measure of individual differences in the effect of competition across participants (McClure and van den Bos, 2011; van den Bos et al., 2013). In essence, we measure the effect of competition as the amount of money that participants are willing to lose in order to win auctions.

We report two studies that use two distinct approaches to relate competitive drive to social status. First, we manipulated social context in order to increase the salience of social status. Specifically, a large body of work (Akerlof and Kranton, 2010) has shown that the incorporation of identity in economic models can explain behavior that at first appears (economically) detrimental. This work suggests that people have identity-based payoffs derived from their own and other people's actions. For example, men may gain utility from actions that confirm their manhood, but disutility from actions that threaten this identity. Similarly, people may derive utility from actions that impact their perceived status, particularly when social status is highly salient (Immorlica et al., 2012).

Our identities are complex and fluid. As a result, different social contexts emphasize different aspects of our identity. Research from social psychology has shown that minimal group paradigms alter the salience of social comparisons (Brewer and Weber, 1994). The heightened relevance of social comparison may increase the desirability of being perceived as a high-status individual (Ridgeway, 2002; Garcia et al., 2005) and in turn impact social preferences over outcomes (i.e., increased utility for winning and/or increased disutility for losing). In the first experiment we investigated the effect of increased salience of social status by taking advantage of a naturally occurring rivalry between two universities. We contrast bidding when (1) participants believed that out-group members were present in the auction against (2) when participants perform the task in the absence of explicit group identities. We hypothesized that the emphasis on the participants' identity, particularly given the existing competitive relationship targeted by our manipulation (Schloss et al., 2011), would increase the utility gained from obtaining status and hence increase overbidding (Akerlof and Kranton, 2000). Finally, we explored the role of affective response to social outcomes in relation to the formal analyses of individual differences in social utility.

Our second study takes advantage of the fact that differences in basal testosterone levels predict the drive for social status, both across individuals and within individuals across time (Mazur and Booth, 1998; Mehta et al., 2008; Eisenegger et al., 2011). Additional evidence indicates that people with high basal testosterone levels experience pleasure or dysphoria when they succeed or fail to achieve higher status, whereas low testosterone individuals show no such affective responses to status changes (Josephs et al., 2003; Newman et al., 2005; Mehta et al., 2008). We hypothesized that basal hormone levels would influence affective responses to status changes inherent in our auction task, and hence would be associated with increased overbidding. We test this prediction in a second experiment.

Overall, we argue that competitive drive arises from a desire to obtain or maintain social status, giving rise to behaviors that may have negative financial consequences. We conclude that competitiveness is strongly driven by emotions arising from social comparison and that economic theory ought to incorporate motivations related to social context and status.

## Experiment 1: stanford vs. berkeley

### Method

#### Participants

We recruited 47 male participants from a paid participant pool maintained by the Stanford University Psychology Department. The control group consisted of 21 participants (mean age = 25.59 years, *SD* = 10.90) after excluding 6 who did not believe the cover story. The experimental group was composed of 19 subjects (*M* = 21.15 years, *SD* = 4.36); one participant was excluded because of prior experience in a sealed bid auction experiment. The study was approved by the Stanford University Institutional Review Board and all participants gave written, informed consent before completing the task.

#### Sealed bid common value auction

In order to test predictions of the model on competitive behavior, participants played multiple rounds of a 5 player sealed bid auction task. At the start of the experiment, each group of 5 participants received a 15 min tutorial on the auction task using a standardized PowerPoint presentation (see van den Bos et al., 2008, 2013 for details). During the tutorial the following points were explained: (1) the structure of a first price sealed bid common value auction, (2) how to place bids using the computer interface, and (3) the exchange rate between monetary units (MUs) in the game and pay-off in real dollars at the end of the experiment. To ensure comprehension of the task, all participants completed a questionnaire that tested task comprehension before continuing on to the experiment.

In each auction round of the auction task, participants were given independent estimates of the value of an item under auction (*x_i_*, where *i* indexes individual participants), and were provided with the error term (ε) for that round. Subjects knew from the tutorial that estimates were drawn from a uniform distribution with maximum error ε around the true, but unknown, common value (*x*_0_) of the item under auction. During the tutorial, the difference between a normal and a uniform distribution was explained, and it was emphasized that any estimate (*x_i_* greater or less than, but within ε of *x*_0_) was equally likely. The error term ε was the same for all participants in each round, but changed between rounds (ε ~ {4, 5, 6}). The true value, *x*_0_, was randomly drawn from a uniform distribution with lower and upper bounds of *x_L_* = 10 MUs and *x_U_* = 75 MUs. As described in van den Bos et al. (2008) we used a different distribution when selecting true values (*x*_0_ ∈ [*x_L_* + ε_max_ to *x_H_* − 2ε_max_]) to ensure that the optimal bid could be calculated by *x_i_* − ε (see Methods below). In sum, participants were informed that the true value (*x*_0_) was picked from the uniform distribution ([*x_L_, x_H_*]), and that they would only be given an estimate (*x_i_*) of this true value and the error (ε) in order to determine how to bid.

**Figure 1:**
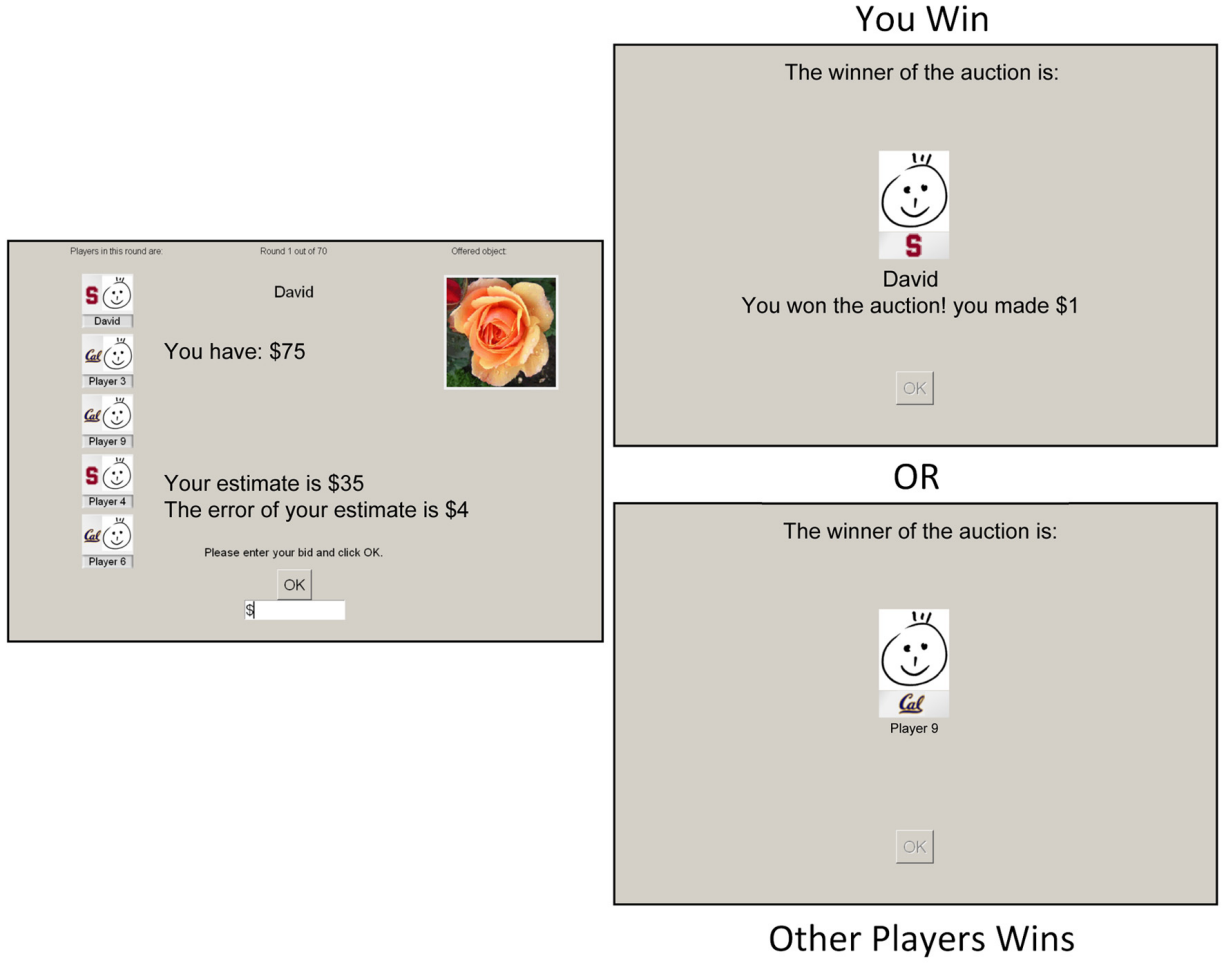
**Common value auction experiment design**. Each round a new object (flower) was presented with an personal estimation of the value and error term indicating how much their estimation may differ from the real value. After all bids were submitted, the outcome was shown at a variable delay of 4–8 s. Finally, a 6 s display showed either the winner of the auction, or the amount of revenue gained or lost if the participant was the winner herself. This is an example of the Stanford vs. Berkeley version. In the real experiment we have used real names and pictures of the other players and the participant.

After all players submitted their bid based on this information, the highest bid was determined and the winner's picture was shown to all players (see Figure 1 for a detailed timeline and example stimuli). Only the winner gained information about the true value of the object and the revenue made in that round. Revenue was determined by *x*_0_− *b*_max_ and was negative when the winning bid (*b*_max_) was larger than true value *x*_0_.

**Figure A1:**
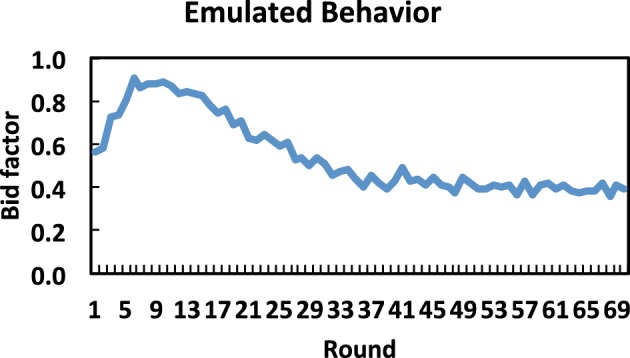
**Average bid factor of 35 participants that participated in a pilot study with a 5-player auction with real players**. For each computer player a bid factor was picked from a Gaussian distribution of 0.2 around the average bid factor.

The experiment consisted of seventy consecutive sealed bid auctions. For both the control and experimental groups, a cover story was used to make the participants believe they were playing against other human opponents, while in reality the other players were simulated by a computer algorithm (cf. van den Bos et al., 2008). For every round of the task, computer bids for four simulated participants were derived from predefined bidding strategies that were based on the result of a pilot study (*N* = 35, see Figure A1) in which participants did play with real other players. After completing the last auction, participants were debriefed and asked about their belief regarding the multi-player nature of the experiment. Participants who did not fully believe that they were bidding against other people were excluded from data analysis. The experiment took about 45 min to complete.

#### Experimental manipulation

Before participating in the study, participants were sent multiple emails emphasizing the importance of arriving on time because of the multi-player nature of the experiment. On the day of the experiment, a picture was taken of the participants to be used during the auction task. In the experimental condition, the participants were instructed that this experiment was part of a larger study in collaboration with UC Berkeley. This was explained in neutral terms, to minimize differences from the control condition. The only substantial difference across conditions was that, in the experimental condition, each player was represented with her own picture *and* the logo of the university she was attending (see Figure 1).

#### Behavioral analyses

Based on the signal (*x_i_*) and the error (ε), the (optimal) risk-neutral Nash equilibrium (RNNE) bidding strategy can be determined for each round and each participant. The solution is given by:

(1) $$\text{RNNE}=x_{i}-\varepsilon +Y,$$

Where

(2) $$Y=\frac{2\varepsilon }{n+1}\text{exp}\left(\frac{n}{2\varepsilon }\left[x_{i}-\left(x_{L}+\varepsilon \right)\right]\right),$$

*n* is the number of bidders, and *i* indexes participants (Kagel and Levin, 2009). Following our previous study (van den Bos et al., 2008), we selected values of *x*_0_ so that the term *Y* from Equation 2 is almost zero and can thus be safely ignored. As a result the RNNE strategy is reduced to the equation:

(3) $$\text{RNNE}=x_{i}-\varepsilon ,$$

We analyzed behavior using a term that expresses bids relative to this optimal strategy. Over/under-bidding relative to the error ε is summarized by the bid factor, κ:

(4) $$\kappa =\frac{b_{i}-(x_{i}-\varepsilon )}{\varepsilon },$$

were *b_i_* is the bid the participant submitted based on signal *x_i_*. A bid factor of 1 implies that a participant's bid, *b_i_*, is equal to her signal *x_i_*, whereas a bid factor of 0 approximates RNNE.

#### Reinforcement-learning model

Following our prior work (McClure and van den Bos, 2011; van den Bos et al., 2013), a reinforcement learning model was used to summarize and interpret bidding during the task. The model assumes that subjective value depends on both monetary revenue (i.e., *x*_0_ − *b_i_* for the winning bidder and 0 for others) as well-separate utility parameters associated winning (ρ_win_) and not winning (ρ_loss_) an auction. Thus, after winning an auction, value was assumed to equal the monetary revenue plus the utility of winning, ρ_win_. By contrast, after losing, value is determined solely by the magnitude of (individually determined) disutility of not-winning ρ_loss_:

(5) $$U_{i}=\{\begin{array}{l} x_{0}-b_{i}+\rho_{\text{win}}\text{if }b_{i}=\max(b)\\ -\rho_{\text{loss}}\text{ otherwise} \end{array}$$

For the reinforcement learning model we assumed that, at the end of every round, a prediction error (δ_*i*_) was calculated based on the difference between the actual outcome (*U_i_*) and the outcome (*V_i_*) expected by bidding a given bid factor (κ_*i*_):

(6) δ(κ)=U(κ)−V(κ)

For simplicity, we omit the subscript *i* that indexes participants in Equation 6 for the remainder of the paper. This prediction error was used to update the estimated value associated with different bidding strategies (*V*(κ)). Note that through learning *V*(κ) will converge to the expected value of bidding a certain bid factor, includes both the monetary payoffs as well as the utility of winning and losing, ρ_win_ and ρ_loss_. Because κ is a finely discretized variable, the number of states over which it is necessary to learn state-action values is very large. For modeling purposes, we restricted predicted behavior to the approximate range of bid factors submitted by participants in the experiment: −1 to 2, discretized in steps of 0.01. Furthermore, we assumed that participants inferred that (1) when winning, larger bids would have also won, although with less net monetary utility, and (2) when losing, smaller bids would have also lost. This assumption allowed us to update a range of value estimates, for values of κ greater than or less than that submitted, on each round of the auction (McClure and van den Bos, 2011; van den Bos et al., 2013).

Learning based on reward prediction errors is modeled as in most RL methods, with a learning rate (α) determining the influence of δ on new values of *V*(κ′):

(7) $$V(\kappa \prime )\leftarrow V(\kappa \prime )+\alpha_{\kappa \prime }\delta (\kappa \prime )$$

In the current model we scaled learning rate so that updating only occurs within a limited range of the bid factor employed on any trial in order to account for the fact that the probability of winning with a given bid factor changes over time. This was implemented by creating an effective learning rate that decreases inversely with distance from κ:

(8) $$\alpha_{\kappa \prime }=\frac{\alpha }{1+\kappa \prime -\kappa }$$

Decisions were then generated by the model using a soft-max decision function, with a parameter *m* that modifies the likelihood of selecting bids:

(9) $$P(\kappa )=\frac{\exp\left(mV(\kappa )\right)}{\sum_{\kappa \prime }\exp\left(mV(\kappa \prime )\right)}$$

The value function, *V*, was initialized to zero for all values of κ. The denominator sums over all possible values of κ (indexed by κ′ ∈ [−1, 2] as discussed above). We also experimented with randomized initial values of *V*(κ), which is commonly used in RL algorithms to encourage initial exploration of strategies, however, randomizing initial values did not affect the performance of the model in any notable way (McClure and van den Bos, 2011). All model-related results are reported for fits conducted with *V* initialized to zero. Note that previous model comparisons have indicated that the ρ_win_ and ρ_loss_ parameters are crucial for the model to asymptote at a bid factor κ > 0. A standard learning model without ρ_win_ and ρ_loss_ will necessarily result in an asymptote of κ = 0 (see van den Bos et al., 2013).

We estimated the parameters (ρ_win_, ρ_loss_, α, and *m*) of the RL model using a simplex optimization algorithm in Matlab. The model simulated the performance of five bidders with average bid factors calculated for each round of 70 consecutive auctions in 10000 runs of the model. A similar round-by-round average bid factor was also calculated for the bids submitted by the participants in the study. Best-fitting model parameters were determined at the group level so as to minimize the sum-squared error between average model performance and the average subject performance. Group-based estimates of α and *m* were subsequently used in a second model fitting procedure that was aimed at estimating the individual differences in ρ_win_ and ρ_loss_ for the participants in the Experiment 1.

#### Sequential analyses and social utility

For behavioral analyses we defined two dependent variables to investigate the relationship between model parameters and choice behavior: [Δκ | win] and [Δκ | not win]. These two measures of sequential changes in bid factor (κ) were computed by calculating the average change in κ(κ (*t* + 1) − κ(*t*) following either winning or not winning a round in the auction. To test whether the individually estimated parameters for ρ_win_ and ρ_loss_ predict different aspects of participants' behavior, both estimates were simultaneously regressed against [Δκ | win] and [Δκ | not win] using multiple regression.

#### Affective responses questionnaire

After the experiment, participants were asked to report their affective responses to different social and monetary aspects of auction outcomes (e.g., “Realizing that another player wins a lot of auctions made me feel …,” “ Losing money made me feel … ”; see Table A1). All items were answered using a seven-point Likert scale ranging from “very negative” to “very positive.” Factor analyses yielded two factors: a monetary and a social factor (Cronbach's α = 0.71 and 0.76, respectively; for more information see Figure A1 and (van den Bos et al., 2013). The non-weighted mean scores on the monetary and social items were used as predictors for individual differences in competitive behavior.

### Results

**Figure 2:**
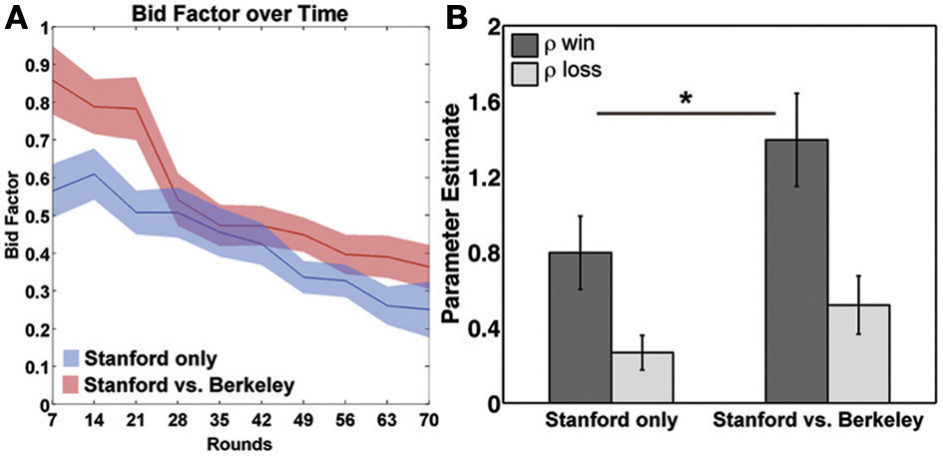
**(A)** Development of the bidfactor over time and **(B)** parameter estimates of the utility of winning and losing. ^*^p < 0.05.

The goal of this experiment was to test whether the competitiveness of the social environment influences overbidding. We therefore performed a repeated measures ANOVA with time (grouped into bins of 10 consecutive rounds of actions) as a within-participant factor and context (experimental vs. control) as a between-participant factor for the average bid factor (κ) across participants. As expected, there was a main effect of time, indicating that participants learned to bid closer to the optimum as the experiment progressed [*F*_(9, 30)_ = 18.08, *p* < 0.001, see Figure 2A]. There was also a significant main effect of experiment condition, with participants in the Stanford/Berkeley context bidding with a significantly higher bid factor than those in the control condition [*t*_(38)_ = 1.85, *p* < 0.03, one-tailed]. There was no interaction between time and social context, indicating that both groups learned to improve their bids at comparable rates [*F*_(9, 30)_ = 1.81, *p* = 0.12].

Based on visual inspection of the data (Figure 2A) we performed *post-hoc* tests of the last for blocks of the task in order to test whether differences in bidding were present at the end of the task across conditions. These analyses revealed that there was no longer a main effect of time, indicating that participants bidding strategy was stabilizing [*F*_(3, 30)_ = 1.12, *p* = 0.3]. However, there was a significant main effect of condition [*F*_(3, 30)_ = 2.94, *p* < 0.03], with participants in the Stanford/Berkeley context bidding with a significantly higher bid factor than those in the control condition.

One limitation of the above analysis is its insensitivity to idiosyncratic differences in bidding and win/loss history of each participant. Moreover, grouping auctions into bins of 10 rounds may obscure differences in how social context influences the way that participants respond to winning and losing against different competitors. To overcome these problems, we fit a reinforcement learning model to the subjects' round-to-round behavioral data. This produced estimates of the value of winning and losing, independent of monetary outcomes, for each participant. We refer to the utility of winning and losing as ρ_win_ and ρ_loss_, respectively. Since ρ_win_ and ρ_loss_ are assumed to influence the subjective value of different auction outcomes, the parameters should correlate with how people adjust their bidding round-to-round, independent of monetary outcomes. We tested for this relationship by regressing ρ_win_ and ρ_loss_ against changes in bidding (Δκ) following a win or non-win, respectively. A multiple robust regression, with Huber weighting function, of both ρ_win_ and ρ_loss_ on [Δκ | win] fitted significantly [*r* = 0.45, *F*_(2, 40)_ = 4.47, *p* < 0.02], but only ρ_win_ [β = 0.69, *t*_(40)_ = 3.84, *p* < 0.001] and not ρ_loss_ [β = −0.13, *t*_(40)_ = −0.62, *p* = 0.54] contributed significantly to the regression. In contrast, in the regression against [Δκ | non-win] [*r* = 0.46, *F*_(2, 40)_ = 4.74, *p* < 0.02], ρ_loss_ contributed significantly [β = 0.30, *t*_(40)_ = 2.75, *p* < 0.02], but not ρ_win_ [β = −0.16, *t*_(40)_ = −0.63, *p* = 0.48].

Both of the social utility parameters, ρ_win_ and ρ_loss_, were significantly greater than zero in both experimental groups (*p* < 0.01 for all one-sample *t*-tests; see Figure 2B). The fact that social factors influence bidding replicates our previous findings (van den Bos et al., 2008, 2013). Our primary interest here was in determining whether emphasizing the social identity in the auction increases ρ_win_ and ρ_loss_. To this end, we found that ρ_win_ was significantly greater in when in the Stanford/Berkeley condition relative to control [*t*_(38)_ = 1.9, *p* < 0.03, one tailed see Figure 2B]. Additionally, ρ_loss_ showed a trend for being larger in the presence of Berkley students [*t*_(38)_ = 1.42, *p* = 0.08, one tailed].

Our design also allowed for the further exploration of within-subject effects in the Stanford/Berkley auction. In particular we were interested in whether the presence of Berkeley students had a general effect on overbidding, as the results above suggests, or whether overbidding was dependent on the number of Berkeley players present in the auction. We found no evidence of a relationship between bidding and the number of Berkley players in the auction (*r* = 0.01, *p* = 0.9). Taken together, these results support the hypothesis that a more status-salient context may lead to a general increase in overbidding because of its effect on magnifying the social utility attributed to outcomes, particularly the social utility of being the winner.

The above analyses show that ρ_win_ and ρ_loss_ had dissociable effects on competitive bidding strategies in the auction task that varied by social context. To further explored the nature of ρ_win_ and ρ_loss_, we correlated individually determined parameter estimates with self-reported measures of affective responses to auction outcomes in both groups. The results of these analyses showed that individual differences in both ρ_win_ and ρ_loss_ are directly related to feelings associated with the social impact of winning or losing an auction (Spearman's ρ = 0.47, *p* < 0.003 and Spearman's ρ = −0.36, *p* < 0.03, respectively). By contrast, ρ_win_ and ρ_loss_ were not related to preferences over monetary gains and losses (Spearman's ρ = −0.16, *p* = 0.32 and Spearman's ρ = −0.22, *p* = 0.18, respectively). *Post hoc* comparison of correlation coefficients also revealed that the absolute correlations of ρ_win_ and ρ_loss_ with the social factor were significantly larger than with the money factor (*z* = 2.88, *p* < 0.001 and *z* = 3.11, *p* < 0.001, respectively).

Taken together, these results indicate that bidding in common value auction is sensitive to social context such that overbidding increases when the social utility and affective responses attributed to outcomes is elevated.

## Experiment 2: testosterone and cortisol

### Method

#### Participants

Twenty-six white, right-handed, male participants were recruited for the study (mean age 24.11 years, *SD* = 10.35). Ethnicity and gender were restricted to account for known differences in basal testosterone levels. Participants played seventy rounds of a five player sealed bid auction; task procedures were the same as above. A cover story led the participants to believe they were playing against other human opponents present at Stanford University, while in reality the other players were simulated by the computer. As part of the cover story, participants received multiple e-mail reminders ahead of the experiment indicating that they should be on time because they would participate in a multi-player on-line auction. Three participants were excluded from data analysis because they did not believe the cover story. The study was approved by the Stanford University Institutional Review Board, and all participants gave written informed consent before completing the task.

#### The expert auction

The expert auction uses the same common value auction and experimental procedures as in Experiment 1. However, in this version, participants were taught how to bid using the optimal RNNE strategy prior to beginning the experiment (see Equation 3). All participants completed a questionnaire before the experiment to ensure comprehension of the task and the RNNE strategy. Everyone completed this questionnaire without error. In order to match the bidding strategies of the simulated players, the computer bids were based on the behavior of expert participants from a previously published study (van den Bos et al., 2008, Experiment 2). Furthermore, in this version of the task the number of auctions won by each player was displayed on the screen.

#### Behavioral analyses

As in Experiment 1, we used the bid factor κ to measure overbidding. Recall that a bid factor of 1 implies that participants bid their estimate (*x_i_*) of the true value (*x*_0_), whereas a bid factor of 0 indicates bidding RNNE. In this experiment, positive values for κ occur when participants knowingly and willingly overbid since all participants knew the optimal bidding strategy from the outset of the task.

#### Testosterone

Testosterone is well-established to promote behaviors to seek or protect social status in the face of competition (Mazur and Booth, 1998; Eisenegger et al., 2011). We collected two saliva samples in order to measure individual differences in basal testosterone. The first saliva samples were collected from participants immediately upon arrival after obtaining written consent, and were immediately frozen below −20°C. The second saliva samples were collected and at the end of the experiment. Participants were informed that their saliva would be used to estimate testosterone and cortisol levels. Saliva assays were obtained using Salimetrics Oral Swabs, following standard protocol. All participants were tested during the same time period, 4:00–4:45 and 5:15–6:00 pm, to account for circadian changes in endocrine levels.

Serum testosterone and cortisol concentrations measured before and after the test were positively correlated across all of the subjects (*r* = 0.89, *p* < 0.001 and *r* = 0.85, *p* < 0.001, respectively). To reduce noise inherent to the salivary assessments, we therefore used the average concentration in the pre-test and the post-test sample as our independent variable. Several studies have shown that the relationship between testosterone and dominance is moderated by the major human stress hormone cortisol (Dabbs, 1990; Popma et al., 2007; Mehta and Josephs, 2010). We therefore measured salivary concentrations of both testosterone and cortisol. Linear regression analyses were performed with each participant's mean bid factor κ as the dependent variable and with testosterone, cortisol, and testosterone × cortisol as independent variables. All variables in the regression models were standardized, and the interaction term was constructed from standardized values. An additional simple slope analysis was performed to investigate the direction and significance of the relationship between testosterone and overbidding at different levels of cortisol (Popma et al., 2007). Regression analysis for testosterone and bid factor was then performed on a median split of cortisol values.

Finally, we measured a proxy of prenatal testosterone, the ratio in the lengths between the second and fourth fingers (2D:4D ratio). This ratio has been shown in some studies to predict the effects of testosterone on social behavior (Coates et al., 2009a; Brañas-Garza and Rustichini, 2011; Van Honk et al., 2011). However, 2D:4D did not show any significant statistical effects in our dataset and is therefore omitted from further discussion.

#### Questionnaires: social comparison, status, and risk

As in Experiment 1, participants were asked to report their affective responses to different social and monetary aspects of auction outcomes. To further establish the relationship between affective responses to social aspects of the auction task and status seeking we used the Flynn questionnaire, which measures individuals' need for social status (Flynn etal., 2006). As expected, our analyses showed a strong correlation between the (reverse scored) Flynn questionnaire and the affective responses to social comparisons (*r* = 0.56, *p* < 0.006) but not monetary outcomes (*r* = −0.16, *p* = 0.46). Again, the non-weighted mean scores on the monetary and social items were used as predictors for individual differences in competitive behavior.

Finally, given that individual differences in financial risk attitudes have been associated with both basal testosterone levels (Apicella et al., 2008; Coates et al., 2009b) and overbidding (Holt and Sherman, 2000), participants completed the DOSPERT30 (Blais and Weber, 2006) to assess and account for individual differences in financial risk taking. Individual differences in risk preferences were added as a covariate to the regression model testing for the relation between testosterone, cortisol and bidding behavior.

### Results

**Figure 3:**
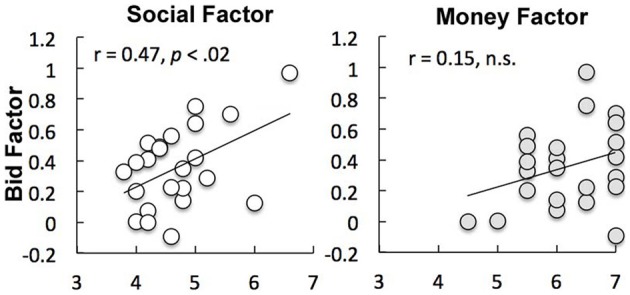
**Interaction between testosterone and cortisol in relation to overbidding**. A significant positive relationship between testosterone and overbidding was found at low cortisol but not low cortisol values. The x-axis represents *z*-transformed testosterone levels.

Replicating earlier findings (van den Bos et al., 2008), we found that even though participants were fully aware of the RNNE strategy, they still overbid significantly [mean κ = 0.36, *SD* = 0.26, *t*_(22)_ = 6.45, *p* < 0.001], which resulted in an average loss of 9.78 MUs [*t*_(22)_ = −2.30, *p* < 0.03] over the course of the experiment. A robust linear regression model predicting overbidding from basal testosterone and cortisol levels was significant [with Huber weighting function (Venables and Ripley, 2002); *r*^2^ = 0.614, *F*_(4, 18)_ = 5.68, *p* < 0.006]. See Table 1 for the full regression results and Table 2 for an overview of descriptive statistics and correlations between variables. For overbidding, a significant effect of testosterone [β = 0.47, *t*_(18)_ = 2.16, *p* < 0.04] and testosterone × cortisol [β = −0.80, *t*_(18)_ = −3.19, *p* < 0.005] was found, while the effects of cortisol [β = −0.34, *t*_(18)_ = −1.78, *p* = 0.09] and risk attitude [β = 0.16, *t*_(18)_ = 0.80, *p* = 0.44] were not significant. To further study the interaction, simple slope analyses were performed on median split by cortisol level (see Figure 3). A significant slope was found in the low cortisol group [β = 0.65, *t*_(11)_ = 2.61, *p* < 0.02], reflecting a significant positive association between testosterone and overbidding at this level of cortisol. No effect was found in the high cortisol group [β = 0.32, *t*_(10)_ = 1.52, *p* = 0.14]. In sum, we found that testosterone predicted overbidding, particularly for the group with low levels of cortisol.

**Table 1:** **Robust linear regression model predicting overbidding**.

	B^*^	**t**	***p***
Testosterone	0.47	2.15	0.04
Cortisol	−0.34	−1.77	0.09
Testosterone × Cortisol	−0.80	−3.18	0.005
Risk	0.15	0.79	0.44

* Standardized coefficients.

**Table 2:** **Correlations among variables**.

		**I**	**II**	**III**	**IV**
I	Bid Factor (*k*)				
II	Testosterone	0.48^*^			
III	Cortisol	0.08	0.32^*^		
IV	Risk	0.09	0.26	0.29	
V	Social comparison	0.56^**^	0.42^*^	−0.21	−0.05

** p < 0.001,

* p < 0.05.

**Figure 4:**
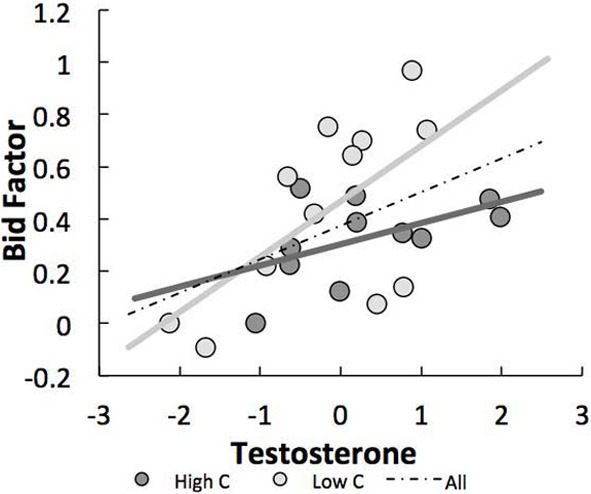
**Individual differences in levels of overbidding were correlated with self-report measures of affective responses to social (*r* = 0.47, *p* < 0.02) but not monetary (*r* = 0.15, *p* = 0.31) outcomes**.

The analyses of the questionnaire indicated that participants cared about both the social and the monetary outcomes of the auctions [mean absolute rating of importance on 7-point Likert scale = 4.9, σ = 0.7, *t*_(22)_ = 29.87 against the null hypothesis of “not-important” rating of 4, *p* < 0.001 and μ = 5.0, σ = 0.7, *t*_(22)_ = 66.61, *p* < 0.001 for social and monetary items, respectively]. However, individual differences in mean levels of overbidding during the experiment (mean κ) were correlated with self-report measures of affective responses to social comparisons (*r* = 0.47, *p* < 0.02) but not monetary outcomes (*r* = 0.15, *p* = 0.31, see Figure 4). *Post-hoc* comparison of *z*-transformed correlation coefficients revealed that these correlations were significantly different (*z* = 2.41, *p* < 0.01).

**Figure 5:**
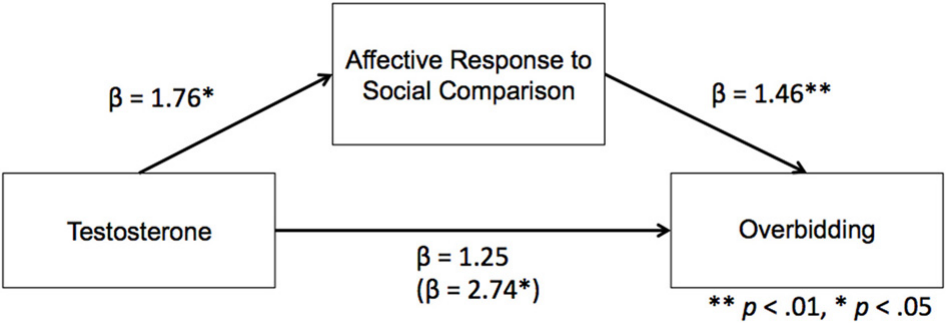
**Mediation of testosterone, affective responses to social comparison and competitive overbidding**. For illustration purposes we have added the betas of the classical (Baron and Kenny, 1986) regression method to test for mediation. The results, which are completely consistent with the bootstrapping methods reported in the results section, show that the relation between testosterone and competitive overbidding is mediated by affective responses to social comparisons. For more detail on moderation effects of cortisol see results.

To further investigate the relationship between testosterone, cortisol, affective responses and competitive bidding, we performed a moderated mediation analysis. Specifically, we tested whether the effect of testosterone on the bid factor was mediated by the self-reported affective responses to social comparison. Based on our simple slope analyses, we expected that the indirect effect would be moderated by levels of cortisol. More specifically we tested whether the relationship between testosterone and affective responses related to social comparisons was conditional on levels of cortisol (see Figure 5).

In order to test the moderated mediation analyses hypothesis we conducted the procedure proposed by (Preacher et al., 2007), using the PROCESS algorithm provided by Hayes (Hayes, 2012). We calculated the 95% bias corrected bootstrap confidence intervals (CIs) of the indirect effect on the basis of 5000 bootstrap samples. When the CI ranges does not include zero this is considered support for a significant mediation effect. We used the mean as well as a standard deviation above and below the mean cortisol levels to represent Moderate, High, and Low values for the moderation effect, respectively. The 95% CI around the indirect effect ranged from 0.11 to 0.29 for the Low (−1 SD), 0.05 to 0.22 for the Moderate, and from −0.12 to 0.13 for the High (+1 SD) cortisol group. These results show that the relationship between testosterone and overbidding was not mediated by affective responses related to social comparisons for the High cortisol group. However, the mediation was significant for the Moderate and Low group, supporting the moderated mediation analyses.

Consistent with previous studies, we found support for the dual-hormone hypothesis (Mehta and Josephs, 2010) by showing that the relation between testosterone and competitive behavior is particularly strong when cortisol is low, and not significant when cortisol levels are high. Furthermore, these results suggest that the effect of testosterone on overbidding is mediated by affective responses to social comparisons.

## General discussion

This paper shows that the extent to which participants overbid in a competitive environment is related to two independent measures of drive for social status. First, overbidding was increased by emphasizing a competitive aspect of the participants' social identity. Second, overbidding was predicted by basal levels of testosterone, a hormone strongly associated with the drive for status in humans and animals (Sapolsky, 2004). Thus, both a person's identity, of which the environment may cue particular aspects, and individual differences in biomarkers associated with the drive for status predict costly competitive behavior. As such, these results support the hypothesis that humans not only compete in order to acquire goods but also to establish social status. Furthermore, our results suggest that affective responses, rather than cognitive skill, play an important role in competitive behavior. Taken together, these results suggest that the utility of status gains is partly determined by the biological make-up, and partly by social identity, which in turn is thought to be determined by both the individual and environment factors (Akerlof and Kranton, 2010).

It still remains to be determined precisely what the underlying mechanisms are that may lead social identity or hormones levels to result differences in overbidding. In line with models of anticipated affect (Mellers et al., 1997; Zeelenberg et al., 2000). The correlation between our self-report measure of affect and the ρ_win_ and ρ_loss_ parameters of the reinforcement learning model suggest that the decisions might be determined by both anticipated and experienced outcomes. In a recent study we showed that competitive drive to win auctions is manifest in fMRI BOLD responses in brain reward areas, including the ventral striatum (VS) and ventromedial prefrontal cortex (vmPFC), both strongly associated with the computation of expected and experienced reward value (van den Bos et al., 2013). In particular, responses in the VS and vmPFC reflected both trial-by-trial variations in monetary as well as inferred social prediction errors (see also Fliessbach et al., 2007). Furthermore, we have found that the anterior insula (AI) and temporo-parietal cortex (TPJ) were associated with individual differences in overbidding. Critically, it was not just the level of activity in the AI and TPJ that predicted individual differences in overbidding, but also the degree of functional connectivity between these regions and the VS and vmPFC. Importantly, the level of connectivity was also correlated with ρ_win_, ρ_loss_, and the affective responses to social outcomes. This suggests that one possible mechanism for the increased competition induced by social identify may be the altered value computation in the vmPFC by increased connectivity with the AI and/or TPJ (Carter et al., 2012; Lin et al., 2012).

Interestingly, several studies have shown that local activity and functional connectivity with the vmPFC are associated with behavioral effects of testosterone (Mehta and Beer, 2010; Bos et al., 2012). It seems reasonable to hypothesize that basal testosterone levels are associated with increased functional connectivity between vmPFC and AI/TPJ. Furthermore, we expect that the testosterone related increased connectivity with the vmPFC results in the increased utility attributed to status gains. More specifically, in contrast with the effect of social identity on ρ_win_, we hypothesize that testosterone will lead to the increased utility of winning (ρ_win_) and the disutility of not winning (ρ_loss_). This hypothesis is supported by more qualitative work on testosterone, which suggests that people with high basal testosterone levels experience both more pleasure when they succeed or displeasure when they fail to achieve higher status compared to low testosterone individuals (Josephs et al., 2003; Newman et al., 2005; Mehta et al., 2008). Finally, one suggested mechanism for the interaction between cortisol and testosterone in the regulation of status seeking may be through specific hormonal effects on connectivity between the limbic regions and the vmPFC (Mehta and Josephs, 2010). Future studies that combine the current auction paradigm with measures of hormones and neural activity across different social contexts may reveal the different mechanisms underlying competitive behavior.

In some situations, such as the auction experiment we used, the motivation for status may result in negative financial outcomes. It seems that such deleterious competitive behavior should not have evolved as a stable trait. However, following Mayr's famous distinction between proximate and ultimate causes (Mayr, 1961), it seems likely that the ultimate cause for these (proximal) behavioral mechanisms is that, over the course of evolution, the drive for status results in increased access to resources and mates in the long run. In that sense the overbidding can be seen as a case of costly signaling (Zahavi, 1975; Mazur and Booth, 1998).

Finally, we point to an obvious limitation of our second study is that it only considered male participants. Both testosterone and competition (Gneezy et al., 2003) are known to have a different effect on men and women. For instance, testosterone increases reactive aggression in men but not women (Josephs et al., 2011). Another important limitation is that we have correlated behavior with basal levels of testosterone and thus cannot make a strong claim about causality. Future studies, focusing on female samples, or use the administration of testosterone, may therefore reveal more details about the complex relations between hormones and competitive behavior. Notwithstanding these limitations, the current findings add to a growing literature revealing the relationship between social and affective processes in complex economic behavior, and specifically our understanding of competitive behavior.

### Conflict of interest statement

The authors declare that the research was conducted in the absence of any commercial or financial relationships that could be construed as a potential conflict of interest.

## Appendix

### Factor analyses of self-report measures

The Kaiser-Meyer-Olkin measure of sampling adequacy was 0.63, above the recommended value of 0.6, and Bartlett's test of sphericity was significant [χ^2^_(36)_ = 121.26, *p* < 0.001], suggesting a factor analysis is appropriate. Both non-grapical solutions to the Cattel's Scree Test, the optimal coordinate and acceleration factor, proposed by Raîche et al. (2013) indicated that 2 components should be retained in a factor analyses. Finally we performed the maximum-likelihood factor analysis as implemented in R, using the promax (oblique) rotation for the factor loading matrix.

**Table A1:** **Self-report measures of affective responses to social and monetary outcomes**.

**Question**	**Social**	**Monetary**
1. Being the winner of an auction made me feel (*R*)	0.54	
2. Losing the auction made me feel	0.68	
3. Losing money in the auction made me feel		0.99
4. Winning money made me feel (*R*)		0.63
5. Realizing that another player wins a lot of auctions made me feel	0.97	
6. Realizing that other players win more auctions than I do made me feel	0.72	
7. Not winning an auction over a long period of time made me feel	0.62	0.56
8. The possibility that other players could make more money than I do made me feel	0.38	
9. The possibility that other players could make less money than I do made me feel (*R*)	0.45	
Variance explained	0.32	0.26
Cronbach Alpha	0.76	0.71

Table reports the eigenvalue of each item. Values < 0.3 are not reported. Items marked with (R) were reverse scored.

All items reached the minimum criterion of having a primary factor loading of 0.3 or above (see Table A1). Item 7 is considered a part of the social factor given that it has a higher eigenvalue. Furthermore, note that items 8 and 9 have rather low eigenvalues, this is most likely due to the fact that, in this experiment, the participants are not able to directly compare monetary outcomes with other players because since that information was not available. The initial eigenvalues showed that the first two factor explained 32 and 26% of the variance, respectively. Internal consistency for each of the scales was examined using Cronbach's alpha. The alphas were acceptable (0.7 < α < 0.8): 0.76 for the first and 0.71 for the second factor.

## References

[B1] Akerlof G. Kranton R. (2010). Identity Economics: How Our Identities Shape Our Work, Wages, and Well-Being. Princeton, NJ: Princeton University Press

[B2] Akerlof G. A. Kranton R. E. (2000). Economics and identity. Q. J. Econ. 115, 715–753 10.1162/003355300554881

[B3] Apicella C. Dreber A. Campbell B. Gray P. Hoffman M. Little A. (2008). Testosterone and financial risk preferences. Evol. Hum. Behav. 29, 384–390 10.1016/j.evolhumbehav.2008.07.001

[B4] Barkow J. H. (1989). Darwin, Sex, and Status: Biological Approaches to Mind and Culture. Toronto, ON: University of Toronto

[B4a] Baron R. M. Kenny D. A. (1986). The moderator-mediator variable distinction in social psychological research: conceptual, strategic, and statistical considerations. J. Pers. Soc. Psychol. 51, 1173–1182 10.1037/0022-3514.51.6.1173 3806354

[B5] Blais A.-R. Pr. Weber E. U. (2006). A domain-specific risk-taking (DOSPERT) scale for adult populations. Judgment Decis. Making 1, 33–47

[B7] Bos P. A. Hermans E. J. Ramsey N. F. Van Honk J. (2012). The neural mechanisms by which testosterone acts on interpersonal trust. Neuroimage 61, 730–737 10.1016/j.neuroimage.2012.04.002 22507228

[B8] Brañas-Garza P. Rustichini A. (2011). Organizing effects of testosterone and economic behavior: not just risk taking. PLoS ONE 6:e29842 10.1371/journal.pone.0029842 22242144 PMC3248440

[B8a] Brewer M. Weber J. (1994). Self-evaluation effects of interpersonal versus intergroup social comparison. J. Pers. Soc. Psychol. 66, 268–275 10.1037/0022-3514.66.2.268 8195985

[B10] Carter R. Bowling D. Reeck C. Huettel S. (2012). A distinct role of the temporal-parietal junction in predicting socially guided decisions. Science 337, 109–111 10.1126/science.1219681 22767930 PMC3563331

[B12] Coates J. M. Gurnell M. Rustichini A. (2009a). Second-to-fourth digit ratio predicts success among high-frequency financial traders. Proc. Natl. Acad. Sci. U.S.A. 106, 623–628 10.1073/pnas.0810907106 19139402 PMC2626753

[B13] Coates J. M. Gurnell M. Sarnyai Z. (2009b). From molecule to market: steroid hormones and financial risk-taking. Philos. Trans. R. Soc. B Biol. Sci. 365, 331–343 10.1098/rstb.2009.0193 20026470 PMC2827458

[B14] Dabbs J. M. Jr. (1990). Salivary testosterone measurements: reliability across hours, days, and weeks. Physiol. Behav. 48, 83–86 10.1016/0031-9384(90)90265-6 2236282

[B17] Eisenegger C. Haushofer J. Fehr E. (2011). The role of testosterone in social interaction. Trends Cogn. Sci. 15, 263–271 10.1016/j.tics.2011.04.008 21616702

[B19] Fliessbach K. Weber B. Trautner P. Dohmen T. Sunde U. Elger C. E. (2007). Social comparison affects reward-related brain activity in the human ventral striatum. Science 318, 1305–1308 10.1126/science.1145876 18033886

[B20] Flynn F. J. Reagans R. E. Amanatullah E. T. (2006). Helping one's way to the top: self-monitors achieve status by helping others and knowing who helps whom. J. Pers. Soc. Psychol. 91, 1123–1137 10.1037/0022-3514.91.6.1123 17144769

[B21] Frank R. H. (1993). Choosing the Right Pond: Human Behavior and the Quest for Status. Oxford: Oxford University Press

[B22] Garcia S. M. Tor A. Bazerman M. H. Miller D. T. (2005). Profit maximization versus disadvantageous inequality: the impact of self-categorization. J. Behav. Decis. Making 18, 187–198 10.1002/bdm.494

[B24] Gneezy U. Niederle M. Rustichini A. (2003). Performance in competitive environments: gender differences. Q. J. Econ. 118, 1049–1074 10.1162/00335530360698496

[B25] Hayes A. (2012). PROCESS: A Versatile Computational Tool for Observed Variable Mediation, Moderation, and Conditional Process Modeling. Available online at: http://www.afhayes.com/public/process2012.pdf

[B26] Holt C. Sherman R. (2000). Risk Aversion And The Winner's Curse. Available online at: http://citeseerx.ist.psu.edu/viewdoc/summary?doi=10.1.1.38.1710

[B28] Huberman B. A. Loch C. H. ONculer A. (2004). Status as a valued resource. Soc. Psychol. Q. 67, 103–114 10.1177/019027250406700109

[B29] Immorlica N. Kranton R. Stoddard G. (2012). Striving for social status, in Proceedings of the 13th ACM Conference on Electronic Commerce, (New York, NY: ACM), 672

[B30] Josephs R. A. Mehta P. H. Carré J. M. (2011). Gender and social environment modulate the effects of testosterone on social behavior: comment on Eisenegger et al. Trends Cogn. Sci. 15, 509 10.1016/j.tics.2011.09.002 21974876

[B31] Josephs R. A. Newman M. L. Brown R. P. Beer J. M. (2003). Status, testosterone, and human intellectual performance: stereotype threat as status concern. Psychol. Sci. 14, 158–163 10.1111/1467-9280.t01-1-01435 12661678

[B32] Kagel J. H. Levin D. (2009). Common Value Auctions and the Winner's Curse. Princeton, NJ: Princeton University Press

[B33] Ku G. Malhotra D. (2005). Towards a competitive arousal model of decision-making: a study of auction fever in live and internet auctions. Organ. Behav. Hum. Decis. Process. 96, 89–103 10.1016/j.obhdp.2004.10.001

[B35] Lin A. Adolphs R. Rangel A. (2012). Social and monetary reward learning engage overlapping neural substrates. Soc. Cogn. Affect. Neurosci. 7, 274–281 10.1093/scan/nsr006 21427193 PMC3304477

[B36] Lin N. (1999). Social networks and status attainment. Annu. Rev. Sociol. 25, 467–487 10.1146/annurev.soc.25.1.467

[B37] Mayr E. (1961). Cause and effect in biology: kinds of causes, predictability, and teleology are viewed by a practicing biologist. Science 134, 1501–1506 10.1126/science.134.3489.1501 14471768

[B38] Mazur A. Booth A. (1998). Testosterone and dominance in men. Behav. Brain Sci. 21, 353–397 10.1017/S0140525X98001228 10097017

[B39] McClure S. van den Bos W. (2011). The psychology of common value auctions, in Neural Basis of Motivational and Cognitive Control, eds Mars R. Sallet J. Rushworth M. Yeung N. (Cambridge, MA: MIT Press) 1–18.

[B40] Mehta P. H. Beer J. (2010). Neural mechanisms of the testosterone–aggression relation: the role of orbitofrontal cortex. J. Cogn. Neurosci. 22, 2357–2368 10.1162/jocn.2009.21389 19925198

[B41] Mehta P. H. Jones A. C. Josephs R. A. (2008). The social endocrinology of dominance: basal testosterone predicts cortisol changes and behavior following victory and defeat. J. Pers. Soc. Psychol. 94, 1078–1093 10.1037/0022-3514.94.6.1078 18505319

[B42] Mehta P. H. Josephs R. A. (2010). Testosterone and cortisol jointly regulate dominance: evidence for a dual-hormone hypothesis. Horm. Behav. 58, 898–906 10.1016/j.yhbeh.2010.08.020 20816841

[B42a] Mellers B. A. Schwartz A. Ho K. Ritov I. (1997). Decision affect theory: emotional reactions to the outcomes of risky options. Psych. Sci. 8, 423–449 10.1111/j.1467-9280.1997.tb00455.x

[B43] Messick D. M. McClintock C. G. (1968). Motivational bases of choice in experimental games. J. Exp. Soc. Psychol. 4, 1–25 10.1016/0022-1031(68)90046-2

[B45] Murphy R. O. Ackerman K. A. Handgraaf M. J. J. (2011). Measuring social value orientation. Judgment Decis. Making 6, 771–781

[B46] Newman M. L. Sellers J. G. Josephs R. A. (2005). Testosterone, cognition, and social status. Horm. Behav. 47, 205–211 10.1016/j.yhbeh.2004.09.008 15664024

[B48] Popma A. Vermeiren R. Geluk C. A. M. L. Rinne T. van den Brink W. Knol D. L. (2007). Cortisol moderates the relationship between testosterone and aggression in delinquent male adolescents. Biol. Psychiatry 61, 405–411 10.1016/j.biopsych.2006.06.006 16950214

[B49] Preacher K. J. K. Rucker D. D. Hayes A. A. F. (2007). Addressing moderated mediation hypotheses: theory, methods, and prescriptions. Multivariate Behav. Res. 42, 185–227 10.1080/00273170701341316 26821081

[B49a] Raîche G. Walls T. A. Magis D. Riopel M. Blais J-G. (2013). Non-graphical solutions for Cattell's scree test. Methodology 9, 23–29 10.1027/1614-2241/a000051

[B50] Ridgeway C. (2002). Gender, status, and leadership. J. Soc. Issues 57, 637–655 10.1111/0022-4537.00233

[B51] Sapolsky R. M. (2004). Social status and health in humans and other animals. Annu. Rev. Anthropol. 33, 393–418 10.1146/annurev.anthro.33.070203.144000

[B52] Schloss K. B. Poggesi R. M. Palmer S. E. (2011). Effects of university affiliation and “school spirit” on color preferences: Berkeley versus Stanford. Psychon. Bull. Rev. 18, 498–504 10.3758/s13423-011-0073-1 21380587 PMC3098359

[B54] van den Bos W. Li J. Lau T. Maskin E. Cohen J. D. Montague P. R. (2008). The value of victory: social origins of the winner's curse in common value auctions. Judgment Decis. Making 3, 483–492 20305741 PMC2841440

[B55] van den Bos W. Talwar A. McClure S. M. (2013). Reinforcement learning and social preferences in competitive bidding. J. Neurosci. 33, 2137–2146 10.1523/JNEUROSCI.3095-12.2013 23365249 PMC6619103

[B56] Van Honk J. Schutter D. J. Bos P. A. Kruijt A. W. Lentjes E. G. Baron-Cohen S. (2011). Testosterone administration impairs cognitive empathy in women depending on second-to-fourth digit ratio. Proc. Natl. Acad. Sci. U.S.A. 108, 3448–3452 10.1073/pnas.1011891108 21300863 PMC3044405

[B57] Veblen T. (2000). The Theory of the Leisure Class: An Economic Study in the Evolution of Institutions. Boston, MA: Adamant Media Corporation

[B58] Venables W. N. Ripley B. D. (2002). Modern Applied Statistics with S. New York, NY: Springer 10.1007/978-0-387-21706-2

[B59] Zahavi A. (1975). Mate selection—a selection for a handicap. J. theor. Biol. 53, 205–214 10.1016/0022-5193(75)90111-3 1195756

[B60] Zeelenberg M. Van Dijk W. W. Manstead A. S. R. vanr de Pligt J. (2000). On bad decisions and disconfirmed expectancies: the psychology of regret and disappointment. Cogn. Emot. 14, 521–541 10.1080/026999300402781

